# Complete genome sequences generated using hybrid Nanopore-Illumina assembly of two non-typical *Avibacterium paragallinarum* strains isolated from clinically normal chicken flocks

**DOI:** 10.1128/MRA.00128-23

**Published:** 2023-09-01

**Authors:** Amro Hashish, Maria Chaves, Nubia R. Macedo, Yuko Sato, Stephan Schmitz-Esser, Daniel Wilson, Mohamed El-Gazzar

**Affiliations:** 1 Department of Veterinary Diagnostic and Production Animal Medicine, College of Veterinary Medicine, Iowa State University, Ames, Iowa, USA; 2 National Laboratory for Veterinary Quality Control on Poultry Production, Animal Health Research Institute, Agriculture Research Center, Giza, Egypt; 3 Department of Animal Science, Iowa State University, Ames, Iowa, USA; 4 Wilson Veterinary Co., Indianapolis, Indiana, USA; Loyola University Chicago, Chicago, Illinois, USA

**Keywords:** genomes, *Avibacterium paragallinarum*, non-typical, infectious coryza, chicken

## Abstract

We report the complete genome sequences of two non-typical *Avibacterium paragallinarum* (AP) strains isolated from chickens in the absence of clinical signs. The availability of these genomes can aid scientists in improving current diagnostics and increase our understanding of AP epidemiology and pathogenicity in chickens.

## ANNOUNCEMENT

*Avibacterium paragallinarum* (AP) is a primary pathogen and causes infectious coryza (IC) disease in chickens (1–3). There has been a recent increase in the incidence of this disease (4–6). However, multiple-layer flocks reported positive quantitative real-time PCR (qPCR) results without any clinical signs or history of the disease, leading to notable confusion in the diagnosis of this disease. We report the complete genomes of two AP strains (designated AP-2 and AG21-0333) isolated from layer flocks with complete absence of any clinical signs; therefore, they were dubbed non-typical AP (ntAP). The first ntAP isolate (AP-2) was isolated in the Iowa State University—Veterinary Diagnostic Laboratory from the infraorbital sinus as previously described (3). Briefly, the skin under the eyes was seared and the infraorbital sinus was swabbed. The swab was streaked on a blood agar plate that was then cross-streaked with a *Staphylococcus hyicus* nrusing colony and incubated at 37°C with 7.5% CO_2_ for 48 h. The second isolate was obtained from the Animal Disease Diagnostic Laboratory (ADDL), Ohio Department of Agriculture. The two isolates were sequenced by both Illumina and Oxford Nanopore Technologies (ONT).

For Illumina sequencing, DNA extraction AP-2 was performed using the MagMAX Pathogen RNA/DNA Kit (Thermo Fisher Scientific, Waltham, MA, USA) as previously described (7). The extracted DNA was used for the preparation of sequencing libraries using the Nextera XT DNA Library Prep Kit (Illumina, USA), generating 300 bp paired-end reads. The sequencing was performed using an Illumina MiSeq system (Illumina, USA). Isolate AG21-0333 was sequenced by ADDL using Illumina MiSeq, and sequences were downloaded from the SRA accession number SRR17662949. This strain (AG21-0333) was sent to ISU to conduct the nanopore sequencing. For ONT sequencing, DNA extraction was prepared using the Circulomics Nanobind CBB Big DNA Kit (Circulomics, Baltimore, MD, USA), the gram-negative bacteria high molecular weight DNA extraction protocol. Nanopore libraries were prepared using the Ligation Sequencing (SQK-LSK109) and native barcoding (EXP-NBD104) kits according to the manufacturer’s protocol (ONT, UK). The library was loaded onto an FLO-MIN106 R9.4.1 flow cell and sequenced with the MinION device (ONT, UK). FastQC v0.11.9 was used to assess read quality for both strains (note: default settings were used for all software unless specified otherwise) (8). Bases below a quality score of 20 were trimmed, and adapter sequences were removed with BBDuk v37.36 using the following options: “ref = adapters.fasta ktrim = r ordered k = 23 hdist = 1 mink = 11 tpe tbo qtrim = w trimq = 20 minlen = 75” (9). The quality-filtered reads were assembled and rotated using Unicycler v0.4.8 within the Bacterial and Viral Bioinformatics Resource Center website (10), using the default parameters. The circularity of the chromosomes (and plasmids, if present) was determined based on the Bandage plots provided by the Unicycler output. All genomes were annotated using NCBI Prokaryotic Genome Annotation Pipeline (PGAP) v.6.3 (11). A summary of the metadata, generated sequences, assembly statistics, and annotation of the genomes is presented in Table 1.

**TABLE 1:** Data associated with the two sequenced ntAP strains

	Parameter	Sequenced AP strains	
		AP-2	AG21-0333
Metadata	Species	*Gallus gallus domesticus* (Layer-Flock)	*Gallus gallus domesticus* (Layer-Flock)
	Age of the flock	109 wk	Not available
	State/Country	Iowa—USA	Ohio–USA
	Date of sample collection	1/20/2021	3/9/2021
	Isolation source	Infraorbital sinus	Choanal swab
Raw sequencing reads	Illumina MiSeq paired-end read length (bp)	250	250
	Number of Illumina MiSeq reads	1,164,803	1,102,752
	Average Illumina MiSeq coverage	199.6	183.3
	Total Illumina MiSeq sequencing data (Mbp)	515.3	447.1
	Number of Nanopore reads	260,239	14,424
	Average Nanopore coverage	551.5	32.8
	Total Illumina Nanopore sequencing data (Mbp)	147.0	109.3
Assembly statistics	No. of contigs	1	3
	Total length of the chromosome (bp)	2,529,853	2,399,548
	Number of detected plasmids	-	2
	Total length of the plasmid (bp)	-	Plasmid 1 = 4,345 Plasmid 2 = 3,548
	GC content (%)	41.00	40.95
	N50 for the hybrid assembly (bp)	2,529,853	2,399,548
Annotation results	Number of CDSs	2,342	2,189
	Number of CDSs (without protein-coding genes)	53	40
	Number of tRNAs	57	58
	Number of rRNAs	19	19
	Number of ncRNAs	4	4
Overall genome-related indices (OGRIs)	**ANIb (%)^*a*^:** To the AP NCBI reference strain ESV-135 NZ_CP050316.1	96.44%	96.41%
	**dDDH^*b*^:** To the AP NCBI reference strain ESV-135 NZ_CP050316.1	71.3%	71%
Virulence genes	***hmtp210* ^*c*^**	Present (19,485 bp)	Present (16,644 bp)
	***ctr*D**	Absent	Absent
	***katA***	Absent	Present
GenBank data	BioSample accession number	SAMN30950991	SAMN30950992
	BioProject accession number	PRJNA882779	PRJNA882779
	Genome assembly accession number	Chromosome CP104917	Chromosome CP104914
			Plasmid-1 CP104915 Plasmid-2 CP104916

^*a*^ Average Nucleotide Identity percentage was calculated based on BLAST+ (12) available via JSpeciesWS online service https://jspecies.ribohost.com/jspeciesws/#home.

^*b*^ Calculated using the online tool available through the GGDC website at https://ggdc.dsmz.de/ “formula 2”.

^*c*^ Length of the *hmtp210* gene from typical AP strains is about 6,100 bp.

Genomic features of the ntAP genomes confirmed their classification as AP; however, the genomic analysis showed meaningful differences from typical AP isolates.

## Data Availability

The genomes are available from NCBI under the accession numbers shown in Table 1.
